# Self-Assembly and Wound Healing Activity of Biomimetic Cycloalkane-Based Lipopeptides

**DOI:** 10.1021/acsami.4c14162

**Published:** 2024-10-18

**Authors:** Anindyasundar Adak, Valeria Castelletto, Ian W. Hamley, Jani Seitsonen, Aniket Jana, Satyajit Ghosh, Nabanita Mukherjee, Surajit Ghosh

**Affiliations:** † School of Chemistry, Pharmacy and Food Biosciences, University of Reading, Whiteknights, Reading RG6 6AH, U.K.; ‡ Nanomicroscopy Center, Aalto University, Puumiehenkuja 2, FIN-02150 Espoo, Finland; § Smart Healthcare, Interdisciplinary Research Platform, Indian Institute of Technology, Jodhpur, Rajasthan 342030, India; ∥ Department of Bioscience and Bioengineering, Indian Institute of Technology, Jodhpur, Rajasthan 342030, India

**Keywords:** peptides, peptide amphiphiles, wound healing, collagen production, self-assembly, aggregation, nanotapes

## Abstract

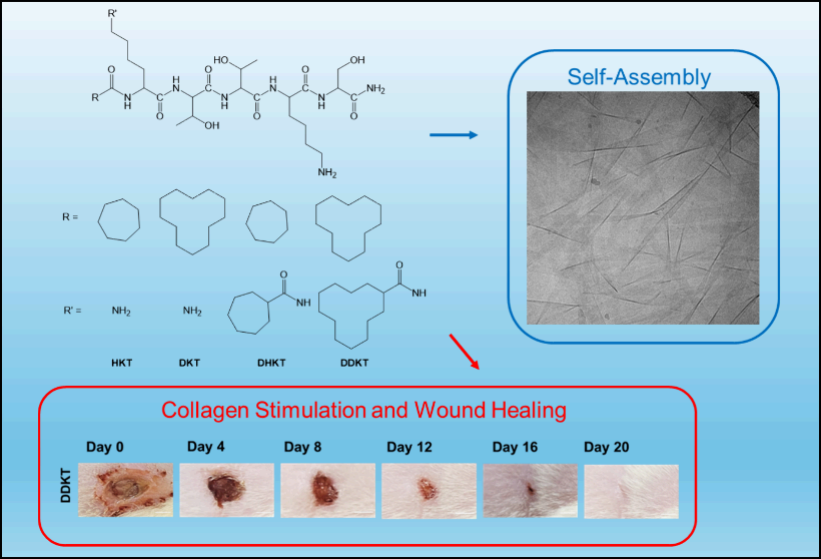

The self-assembly of lipopeptide (peptide amphiphile) molecules bearing single linear lipid chains has been widely studied, as has their diverse range of bioactivities. Here, we introduce lipopeptides bearing one or two cycloalkane chains (cycloheptadecyl or cyclododecyl) conjugated to the collagen-stimulating pentapeptide KTTKS used in Matrixyl formulations. The self-assembly of all four molecules is probed using fluorescence probe measurements to detect the critical aggregation concentration (CAC), and cryogenic-TEM and small-angle X-ray scattering (SAXS) to image the nanostructure. The peptide conformation is studied using circular dichroism (CD) and FTIR spectroscopies. All the cycloalkane lipopeptides show excellent compatibility with dermal fibroblasts. The compounds bearing one or two cyclododecyl chains (denoted as DKT and DDKT, respectively) show wound healing in diabetic rats, the improvement being markedly enhanced for DDKT. Interestingly, the revival of hair follicles and blood vessels in the dermis were observed, which are the critical markers of effective wound repair. Analysis of H&E-stained tissue images (from a rat model) shows that the rat groups treated with DDKT and DKT displayed a significantly increased amount of regenerated hair follicles, indicating a faster healing process for DDKT compared to the control group. Collagen deposition was also enhanced, especially for DDKT, and by day 20, the DDKT-treated groups had developed a dense collagen network accompanied by a regenerated epidermis. At the same time, the number of blood vessels in DDKT-treated diabetic wounds was significantly higher than in control groups and neovascularization was substantially enhanced, as assayed using α-SMA (a marker for vascular smooth muscle cells) and CD31 (a marker specific to vascular endothelial cells). These results suggest that the lead lipopeptide DDKT exhibits a remarkable pro-vascularization capability and shows great promise for future application as a wound-healing biomaterial.

## Introduction

Peptide amphiphiles (PAs) consist of both hydrophobic and hydrophilic segments and belong to an innovative class of biomimetic molecules. Depending on the construction and arrangement of the hydrophobic and hydrophilic regions, they exhibit various self-assembled nanostructures. For example, in surfactant-like peptides (containing linked sequences of hydrophobic and hydrophilic residues), various self-assembled nanostructures can form such as nanofibers,^1,2^ nanotubes,^3^ nanovesicles,^4,5^ etc. In another category, lipidated peptides (lipopeptides, also termed peptide amphiphiles) can form supramolecular nanostructures such as micelles,^6,7^ nanofibers,^8−10^ nanotubes,^100^ etc. The self-assembled nanostructures of these molecules have diverse biomedical applications such as antimicrobials,^11,12^ or in tissue culture,^13,14^ drug delivery,^15,16^ or regenerative medicine.^18,19^

The design and development of biomaterials in regenerative medicine are attracting attention for tissue engineering, tissue repair, and wound healing.^20^ Researchers have explored various strategies in this field over the years. For example, they developed proangiogenic peptide sequences to mimic vascular endothelial growth factor (VEGF) for tissue engineering, repair, and wound healing.^21^ Short peptides have been developed that specifically bind with immunogenic receptors on M2 macrophages, aiming to target anti-inflammatory pathways in order to regulate the immunological response involved in tissue damage and repair.^22^ Another pathway to design and develop pro-adherent sequences is by focusing on designing short peptides such as RGD and RGDS to interact with integrin-binding receptors, which are abundant in various adhesion proteins such as fibronectin and fibrinogen, and structural proteins such as collagen and laminin.^23,24^

In this scenario, it is essential to mimic the native extracellular matrix (ECM), collagen being one of the most crucial proteins in mammals and a major component of connective tissue. Proteolysis of extracellular matrix components leads to the production of short peptides that have been termed matrikines, which have cell signaling properties and are able to influence cell proliferation, migration, and apoptosis.^25,26^ The KTTKS pentapeptide is used in a range of commercial antiwrinkle products, including the palmitoylated (C_16_-hexadecyl-linked) peptide C_16_–KTTKS within the Matrixyl family. Lipidation is used to enhance in vivo stability and potentially enhance skin permeability.^27^ Several reviews on cosmetic peptides are available.^26−30^ The KTTKS pentapeptide sequence is derived from a propeptide of human type I collagen.^31,32^ Interestingly, pentapeptide KTTKS^31^ and its lipidated form C_16_–KTTKS^33^ facilitate the in vitro production of type I collagen. C_16_–KTTKS forms highly extended tape-like nanostructures^34^ and can stimulate type I and type III collagen production in contact with human dermal fibroblast (HDF) cells.^33^ Later, the Hamley group introduced the anionic lipopeptide C_16_-ETTES^35^ and studied its self-assembly into nanotapes as well as its coassembly with C_16_–KTTKS.^35^ This group also introduced its use as a diluent for bioactivity studies,^36^ in mixtures with a lipopeptide bearing an RGD integrin-binding motif, and indeed, the molecule itself was expected to have collagen-stimulating properties.

The development of matrikine-based lipopeptides complements other approaches using functionalized PAs in regenerative medicine, as reviewed elsewhere.^19,37−39^ Examples include heparin-mimetic nanofibers for angiogenesis, laminin- and integrin-based sequences for cell adhesion and spreading, VEGF sequences for angiogenesis and endothelial cell proliferation, diverse sequences to drive cell differentiation into distinct lineages, and many others.^39^ Branched lipopeptides bearing two peptide units or a cyclic peptide have been investigated as self-assembled nanofibril materials,^40^ and lipopeptides containing cyclic peptides are expressed naturally as part of the host defense system of microbes.^41−45^ There are few studies on bioactive lipopeptides with two terminal lipid units,^46−51^ apart from the class of Toll-like receptor agonist lipopeptides.^6,45,52,53^

**Figure 1:**
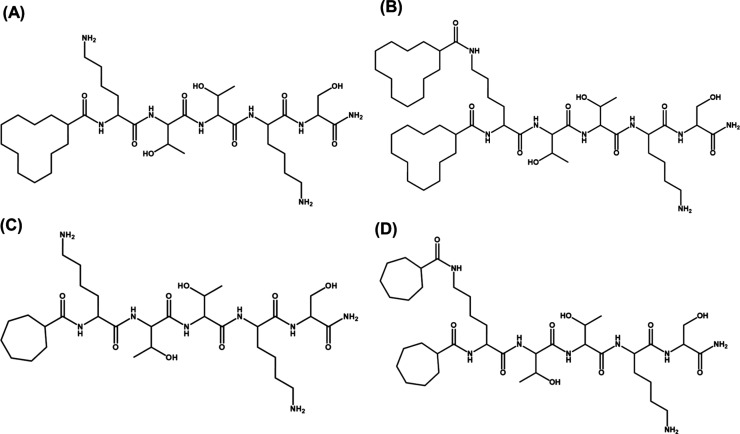
Structure of the cycloalkane-KTTKS peptides (A) single cyclododecane-KTTKS (**DKT**), (B) double cyclododecane-KTTKS (**DDKT**), (C) single cycloheptane-KTTKS (**HKT**), and (D) double cycloheptane-KTTKS (**DHKT**). The C terminal was converted to an amide for all peptides.

Here, we introduce a library of four lipopeptides bearing cyclic lipid chains rather than conventional linear chains attached to the bioactive KTTKS sequence. The molecules are illustrated in Figure 1. They are abbreviated as **DKT, DDKT, HKT,** and **DHKT** for molecules bearing single cyclododecyl, double cyclododecyl, single cycloheptyl, or double cycloheptyl chains, respectively. The cyclic nature of the lipid chains may enable the tuning of molecular packing influencing self-assembly, and potentially bioactivity. It may also enhance in vivo stability, for example reduced degradation by lipases.

After the successful synthesis by solid-phase peptide synthesis (SPPS), the self-assembly of the four lipopeptides was investigated using spectroscopic methods, small-angle X-ray scattering (SAXS), and cryogenic-transmission electron microscopy (cryo-TEM). The conformational properties of lipopeptides in the aqueous solution were examined by using circular dichroism (CD) and Fourier transform infrared (FTIR) spectroscopy. Two different fluorescence probe assays using distinct fluorophores were carried out to determine the critical aggregation concentration (CAC) values of the lipopeptides. Finally, the self-assembled nanostructures were investigated by cryo-TEM and SAXS. The bioactivity of these molecules, especially **DDKT**, in the context of wound healing, was then examined. Cytocompatibility with dermal fibroblasts over a wide concentration range was observed. The wound healing properties were then systematically studied by using a diabetic rat model. Significant dose-dependent collagen deposition was noted. Significantly enhanced wound healing was observed for all four compounds. The analysis of stained tissue sections of the **DDKT** treatment group shows more intact epithelial tissue and elongated hair follicles on the wound surface than the untreated control group. Masson’s trichrome staining shows collagen deposition in the wound healing process. Dense and organized collagen deposition in the **DDKT** treatment group proves its efficacy in the wound healing process. In addition, this lipopeptide promotes vascularization, since after 20 days the number of blood vessels in **DDKT**-treated diabetic wounds is significantly higher than in controls. Immunofluorescence studies show that neovascularization is substantially enhanced, via assays using α-SMA (a marker for vascular smooth muscle cells) or CD31 (a marker for vascular endothelial cells). These results suggest that our lead lipopeptide **DDKT** exhibits remarkable pro-vascularization properties and shows great promise for future application as a wound-healing biomaterial.

## Experimental (Materials and Methods)

### Materials

For lipopeptide synthesis, Rink amide resin, Fmoc-Thr(tBu)–OH, triisopropylsilane (TIS), diisopropylethylamine (DIPEA), Fmoc-Ser(tBu)–OH, Fmoc-cycloalkane-carboxylic acids, O-(1-benzotriazolyl)-1,1,3,3-tetramethyluronium hexafluorophosphate (HBTU), HPLC grade water, and HPLC grade acetonitrile were purchased from Sigma-Aldrich. Fmoc-Lys(Boc)–OH was purchased from Apollo Scientific. Trifluoroacetic acid (TFA) was obtained from TCI. *N*,*N*′-Dimethylformamide (DMF), dichloromethane (DCM), methanol, piperidine, and diethyl ether were purchased from Thermo-Fisher. The purification of the lipopeptides was performed with an Agilent 1200 HPLC with a Supelco C-18 column (Zorbax ODS HPLC Column 15 × 4.6 mm, 5 μm). The solvents used in HPLC were a gradient of water/acetonitrile, where a steady flow rate of 1.2 min/mL was maintained for a run time of 30 min for each cycle.

### Peptide Synthesis

The peptide synthesis was carried out using the solid-phase peptide synthesis (SPPS) procedure where the Rink amide resin was chosen as the solid support. To attach the amino acids and construct the peptides, Fmoc-amino acids (5 equiv), *N*,*N*,*N*′,*N*′-tetramethyl-O-(1H-benzotriazol-1-yl)uronium hexafluorophosphate (HBTU) (5 equiv), and *N*,*N*-diisopropylethylamine (DIPEA) (12 equiv) in DMF were taken as amide coupling agents and purged with nitrogen gas for 6 h. The Fmoc [fluorenylmethoxycarbonyl] group of the amino acids was deprotected by 20% (v/v) piperidine in DMF and purged with nitrogen gas for 30 min. The cycloalkane–carboxylic acid was coupled at the N-terminus of the peptide by the same amide coupling method described above. The synthesized peptides were detached from the resin by adding a solution mixture containing TFA (96%), TIS (2%), and water (H_2_O, 2%), followed by continuous shaking for 4 h at room temperature. The resulting solution was condensed by evaporating TFA using nitrogen gas to a minimum volume and then adding ice-cold diethyl ether to obtain a white precipitate. Next, the white precipitate was collected by centrifugation multiple times and purified by reverse-phase high-performance liquid chromatography (RP-HPLC, Agilent 1200 series). By lyophilization, a solid product was obtained. The purities of the peptides analyzed by HPLC are as follows: **DKT** = 100%, **DDKT** = 99.1%, **HKT** = 99.04%, and **DHKT** = 99.1% (SI Figures S1, S3, S5, and S7). Each lipopeptide was characterized by electrospray ionization–mass spectrometry (spectra shown in SI Figures S2, S4, S6, and S8). The obtained values match with the expected exact molar masses, **DKT**: M_obs_**=** 757 g mol^–1^, M_theo_ = 757 g mol^–1^; **DDKT**: M_obs_ = 951.6 g mol^–1^, M_theo_ = 951.6 g mol^–1^; **HKT**: M_obs_ = 687.4 g mol^–1^, M_theo_ = 687.4 g mol^–1^; and **DHKT**: M_obs_ = 811.5 g mol^–1^, M_theo_ = 811.5 g mol^–1^. The yields are as follows: **HKT** = 71.35%, **DKT** = 68.72%, **DHKT** = 61.91%, and **DDKT** = 58.25%.

### Sample Preparation

To prepare samples of cycloalkane lipopeptides, a measured amount of the solid compound was taken and added to water to make samples with desired concentrations (in wt %) for **DKT** and **HKT**. The pH value of the samples was measured using a Mettler Toledo FiveEasy pH meter with a Sigma-Aldrich micro-pH combination electrode (glass body). The observed pH value was approximately 6.5. Similarly, measured amounts of double cycloalkane lipopeptides were dissolved in 0.1% DMSO/water to obtain the desired concentrations (in wt %) for **DDKT** and **DHKT**. The observed pH value of the samples was approximately 6.5.

### CD Spectroscopy

The CD spectra of samples were measured with a Chirascan spectropolarimeter (Applied Photophysics, Leatherhead, U.K.). For the CD measurements, a 0.1 mm quartz cell was used, spectra being recorded three times in the wavelength range of 280–180 nm. For background correction, the CD spectrum of water was subtracted from the sample spectrum of **DKT** and **HKT,** and 0.1% DMSO/water was used as the background for **DDKT** and **DHKT**.

### Fourier Transform Infrared (FTIR) Spectroscopy

For the FTIR spectral measurements of the cycloalkane lipopeptides, a Thermo-Scientific Nicolet iS5 instrument was used. For each spectrum, 128 scans were recorded over the range of 900–4000 cm^–1^.

### Fluorescence Spectroscopy

The CAC study was performed with a Varian-Cary Eclipse spectrofluorometer. The fluorescence measurements of the samples were recorded using 4 mm inner-width quartz cuvettes at room temperature. For **DKT** and **HKT,** the CAC value was determined with the dye 8-anilo-1-naphthalenesulfonic acid (ANS).^54−56^ Different concentrations of peptides **DKT** and **HKT** were prepared in 2 × 10^–3^ wt % ANS solution (λ_ex_ = 356 nm, from 400 to 650 nm), and their fluorescence spectra were recorded.

To determine the CAC value of **DDKT** and **DHKT,** Thioflavin T (ThT) was used since this dye binds to amyloid fibrils and displays fluorescence.^57,58^ Different concentrations of peptides **DDKT** and **DHKT** were prepared in 5 × 10^–4^ wt % ThT solution (λ_ex_ = 440 nm, from 460 to 650 nm), and their fluorescence spectra were recorded.

By plotting *I*/*I*_0_ versus concentration [in log(wt %)], and determining discontinuities in linear regions, the CAC value was calculated for each peptide, where “*I*_0_” is the peak intensity for the control (ThT solution with no peptide) and ‘*I*’ is the maximum fluorescence intensity of the samples.

### Cryo-TEM

For TEM imaging of the peptides, 1 wt % solutions of the peptides were prepared and 3 μL of the sample was spotted on Quantifoil 3.5/1 holey carbon copper grids (3.5 μm). The TEM images were captured with a field emission cryo-electron microscope (JEOL JEM-3200FSC) equipped with a Gatan Ultrascan 4000 CCD camera. The images were taken in the bright field mode at a temperature of −187 °C.

### SAXS

The SAXS experiments were carried out on the beamline B21 at Diamond (Didcot, U.K.) equipped with a fixed camera length (3.9 m), fixed energy (12.4 keV), and PILATUS 2 M detector. Various concentrations of the samples were prepared and placed in a 96-well plate of an EMBL BioSAXS robot, and each sample was injected via an automated sample exchanger into a quartz capillary (1.8 mm internal diameter) in the X-ray beam. The SAXS data were recorded with 21 frames of 1 s for each sample. Each frame was checked for minimal beam damage, and any anomalous frames were not included in the data averaging. The software ScÅtter was used for data processing.

### Dynamic Light Scattering (DLS)

The distribution of the hydrodynamic radius of the vesicles, *R*_H_, was calculated from the experimental DLS data. DLS data were recorded with a Zetasizer Nano ZS from Malvern Instruments. For each measurement, 120 μL of the sample (0.1 wt %) was placed inside a quartz cell with a 1 mm path length. The DLS data were measured at a fixed scattering angle of 175°. The *R*_H_ distribution was calculated from cumulant fitting of the DLS data using Malvern software.

### Cellular Viability with the MTT Assay

A quantity of 10^4^ human adult dermal fibroblast (HADF) cells was seeded per well of 96-well plates in HiFibroXL Fibroblast Expansion Medium (AL525) for 24–48 h until 60% confluency. The desired concentration of molecules was prepared in the medium and kept for 48 h at 37 °C in a CO_2_ incubator. Then, 5 mg/mL of 3-(4,5-dimethylthiazol-2-yl)-2,5-diphenyltetrazolium bromide (MTT) in media was added in all the wells except the blank well and maintained for 4 h. Finally, the insoluble formazan was dissolved by adding DMSO (100 μL) to each well, and the 96-well plate was placed in a microplate reader, where absorbance (570 nm) was recorded.

### Collagen Deposition Assay

A quantity of 10^5^ HADF cells was seeded per well in 12-well plates in the HiFibroXL Fibroblast Expansion Medium (AL525) for 24–48 h until 60% confluency. The desired concentrations of the molecules were prepared in differentiation media and kept for 72 h for the differentiation. For collagen analysis, the cells were fixed with 4% PFA, and after blocking and permeabilization, the cells were immunostained with the anticollagen 1 antibody (MA1-141,1:250, Invitrogen) and appropriate secondary antibody. Then, the cells were imaged using a fluorescent microscope IX83 equipped with a 40× objective.

### Rat Full-Thickness Wound Model

All animal studies were approved by the Institutional Animal Ethics Committee (IAEC) of JNVU, Jodhpur, India, and conducted following the guidelines of the Committee for the Purpose of Control and Supervision of Experiments on Animals (CPCSEA), New Delhi, India (Approval No: UDZ/IAEC/2023/5). Male albino Wistar rats, weighing 180–195 g and aged 10–12 weeks, were nurtured on a 12 h light–dark cycle and provided with a standard laboratory diet and water ad libitum. To induce diabetes, rats were fasted overnight and administered an intraperitoneal injection of streptozotocin (STZ; Sigma-Aldrich) at a dosage of 45 mg/kg of body weight in 0.1 mol L^–1^ of sodium citrate buffer, pH 4.5. Blood glucose levels were monitored weekly from samples obtained via a tail vein puncture. Rats exhibiting blood glucose levels exceeding 17 mmol L^–1^ throughout the induction period were classified as stably diabetic and subsequently utilized for wound healing experiments. Approximately, 95% of the animals achieved stable hyperglycemia following STZ administration.

Three weeks following the STZ injection, 50 male albino Wistar rats were randomly assigned into five treatment groups: PBS control, **DKT**, **HKT**, **DDKT**, and **DHKT** (*n* = 10 for each group). General anesthesia was maintained through the administration of xylazine and ketamine (Sigma-Aldrich, Germany; 5 mg/kg of body weight xylazine and 40 mg/kg of body weight ketamine, diluted in sterile PBS). During the surgical procedure, a continuous flow of isoflurane (2 mL/min) was used to sustain anesthesia. Before wound creation, the dorsal hair of the diabetic rats was completely shaved and the underlying skin was sterilized with povidone iodine. Full-thickness circular wounds, 8 mm in diameter, were created on one side of the dorsum of the rats by using sterile biopsy punches (Medsor Impex, India). Bioactive peptide solutions were prepared in PBS immediately before their topical application. An amount of 300 μL of 1% (w/v) peptide solution or PBS was applied directly to each wound area. To prevent postsurgical infections and facilitate monitoring, a thin, translucent adhesive film (Tegaderm, 3M, USA) was applied to the wounds. The rats were then returned to their cages for recuperation and were closely monitored for 48 h to detect any early signs of postsurgical stress, secondary infections, or ulcers. At intervals on days 0, 4, 8, 12, 16, and 20, wounds were photographed by using a digital camera set at a fixed distance and magnification. Wound closure was quantified using ImageJ software, applying the formula: % wound closure = [(original wound area – current wound area)/original wound area] × 100%. The weight of the animals was monitored throughout the 20-day experimental period and recorded accordingly.

### Histological Analysis

In the diabetic model, rats were euthanized on day 20 post-wound creation. Skin tissues were fixed in 4% paraformaldehyde and immersed in 30% sucrose for 48 h to achieve cryopreservation. Tissue sections, 5 μm thick, were obtained from OCT-embedded blocks and subsequently analyzed using hematoxylin and eosin (H&E) and Masson’s trichrome staining. The stained sections were then imaged with an Olympus BX-53 upright microscope.

Immunohistochemical staining was performed on 5 μm skin sections from day 20 wounds. The sections were then incubated overnight at 4 °C with primary antibodies: antismooth muscle actin (SMA) (1:500, SC-53142; Santa Cruz, USA) and anti-CD31 (1:500, SC-376764; Santa Cruz, USA). After washing, the sections were treated with secondary antibodies and incubated for 1 h at room temperature. Images were captured at 10× magnification using an Olympus IX83 inverted microscope. The analysis and quantification of the fluorescence micrographs were performed using ImageJ.

## Results and Discussion

We studied the self-assembly, biocompatibility, and wound healing efficiency of the four cycloalkane lipopeptides single cyclododecane-KTTKS (**DKT**), double cyclododecane-KTTKS (**DDKT**), single cycloheptane-KTTKS (**HKT**), and double cycloheptane-KTTKS (**DHKT**) with structures, as shown in Figure 1. The lipopeptides were synthesized following the solid-phase peptide synthesis (SPPS) method using Rink amide resin as a solid support. After the synthesis, the purification of the lipopeptides was performed by reversed-phase high-performance liquid chromatography (RP-HPLC) and they were characterized by mass spectrometry (ESI–MS). From the HPLC and mass spectrometry data, accurate masses and high purities (more than 98%) of the synthesized lipopeptides were observed (Figures S1–S8).

**Figure 2:**
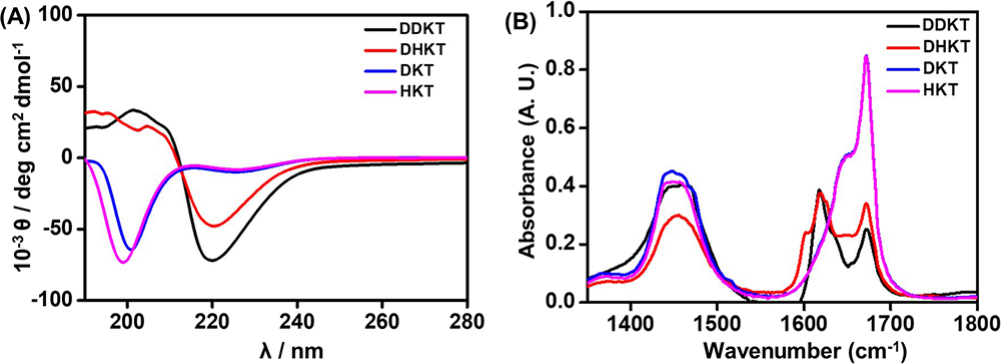
(A) CD spectra of 1 wt % aqueous solution of cycloalkane-KTTKS peptides and (B) FTIR spectra of 1 wt % aqueous solution of cycloalkane-KTTKS peptides.

The conformation of the lipopeptides was examined by using spectroscopic methods. First, the CD spectra of the lipopeptide solutions were recorded to understand the secondary structure of the peptides. The CD spectra for 1 wt % aqueous solutions of **HKT** and **DKT** reveal disordered (random coil) conformations for the single cycloalkane lipopeptides (Figure 2A). Interestingly, for **DHKT** and **DDKT**, the CD spectra exhibit a minimum with a negative band at 218 nm and positive bands near 200 nm, which suggest a β-sheet secondary structure^59−61^ (Figure 2A).

Next, the FTIR spectra for these lipopeptides were recorded in the range of 1320–1740 cm^–1^ to observe the amide-I′ and -II′ regions, which are important to understand the secondary structure of lipopeptides. For **DDKT** and **DHKT**, strong peaks at 1620 cm^–1^ (Figure 2B) were observed in the FTIR spectra, which suggest the formation of a β-sheet secondary structure.^62,63^ However, these peaks are missing for **DKT** and **HKT**, which confirms a random coil structure,^62,63^ consistent with the CD spectra. From SI Figure S9, the peaks corresponding to the CH/CH_2_/CH_3_ stretching modes (at ∼2850 and ∼2920 cm^–1^) of the cycloalkane rings in these molecules were found, which confirm the presence of the cycloalkane chains in the lipopeptides. As clear from Figure 1, both **DDKT** and **DHKT** have an extra cycloalkane ring compared to **DKT**/**HKT**. This leads to higher hydrophobicity of **DDKT**/**DHKT** compared to that of **DKT**/**HKT**, which makes the former more amphiphilic. Due to this, the β-sheet secondary structure plays a crucial role in the self-assembly process of **DDKT**/**DHKT**.

**Figure 3:**
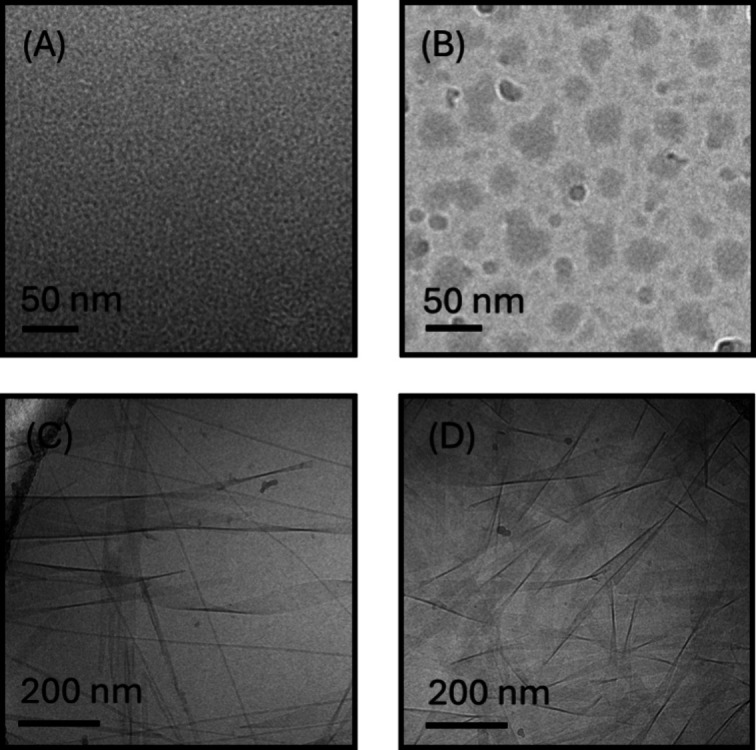
Cryo-TEM images of 1 wt % aqueous solutions of (A) **HKT**, (B) **DKT**, and 1 wt % solutions in 0.1 wt % DMSO/water of (C) **DHKT** and (D) DDKT.

To determine the self-assembled nanostructures of the lipopeptides, cryo-TEM imaging was performed. For the cryo-TEM experiments, 1 wt % aqueous solutions of the samples were prepared for **HKT** and **DKT**, and 1 wt % solutions in 0.1 wt % DMSO/water were prepared for **DHKT** and **DDKT** (which do not fully dissolve in pure water). The cryo-TEM images of the samples **HKT** and **DKT** (Figure 3A,B) reveal globular structures, which were sized by using DLS. This revealed a population of small structures for **DKT** (possibly micelles) as well as polydisperse aggregate structures for both **HKT** and **DKT** with a hydrodynamic radius of approximately 200 nm (SI Figure S10). The cryo-TEM images of the samples **DHKT** and **DDKT** (Figure 3C,D) clearly reveal twisted nanotape structures for both conjugates.

**Figure 4:**
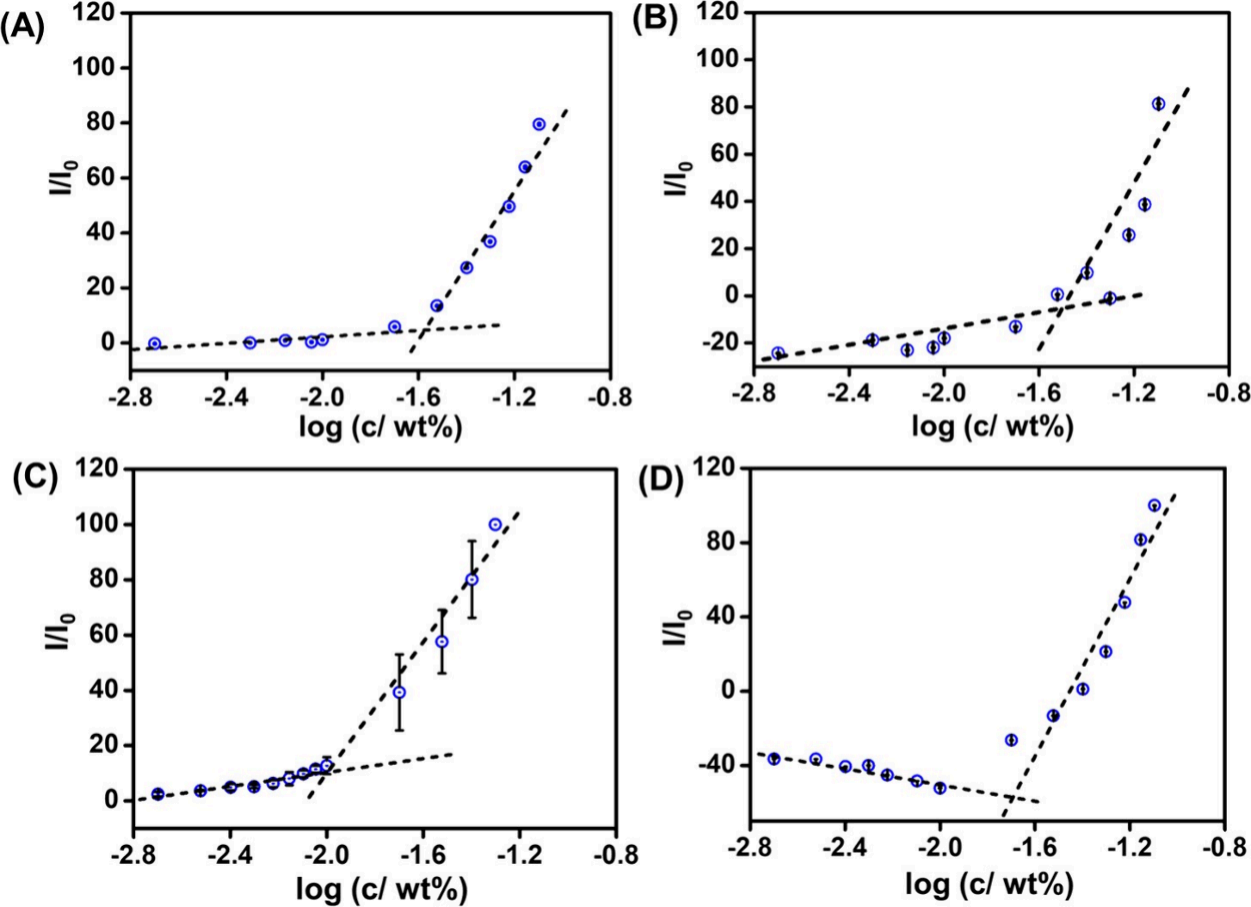
Concentration-dependent dye fluorescence intensity assays to determine the CAC using ANS peak intensity (λ_max_ = 486 nm) of (A) **DKT** and (B) **HKT** or ThT peak intensity (λ_max_ = 487 nm) of peptides (C) **DDKT** and (D) **DHKT**. The error bar corresponds to the standard deviation from the mean (*n* = 3).

Since both **HKT** and **DKT** show globular nanostructures, to determine their CAC values, a fluorescent probe assay using ANS was performed.^56^ After preparing the samples of the peptides at various concentrations with a 2 × 10^–3^ wt % ANS stock solution, emission spectra of the samples were collected (λ_ex_ = 356 nm, λ_max_ = 486 nm). The CAC values were determined from the breakpoint by plotting the fluorescence intensity (*I*/*I*_0_) at 486 nm as a function of the lipopeptide concentration. The CAC was thus determined to be 0.027 ± 0.03 wt % for **DKT** (Figure 4A) and 0.0309 ± 0.05 wt % for **HKT** (Figure 4B). Here, the lower CAC values for **DKT** in comparison to **HKT** result from the higher hydrophobicity of the cyclododecane ring in **DKT** compared to the cycloheptane ring in **HKT**.

Since cryo-TEM shows that **DDKT** and **DHKT** form fibrous nanostructures, to investigate their CAC, a fluorescent probe assay using Thioflavin T (ThT) was performed.^57,58^ After the samples of the peptides were prepared at various concentrations in 5 × 10^–4^ wt % ThT solutions, emission spectra of the samples were collected (λ_ex_ = 440 nm, λ_max_ = 487 nm). The CAC values were determined from the breakpoint by plotting the fluorescence intensity (*I*/*I*_0_) at 487 nm as a function of the concentration. The CAC was calculated to be 0.010 ± 0.03 wt % for **DDKT** (Figure 4C) and 0.032 ± 0.02 wt % for **DHKT** (Figure 4D). Here, the lower CAC value for **DDKT** in comparison to that of **DHKT** is due to the higher hydrophobicity of the double cyclododecane chains in **DDKT** compared to the two cycloheptane rings in **HKT**. Overall, the lower CAC values for **DDKT**/**DHKT** compared to **DKT**/**HKT** are due to greater hydrophobicity and the presence of the β-sheet secondary structure.

**Figure 5:**
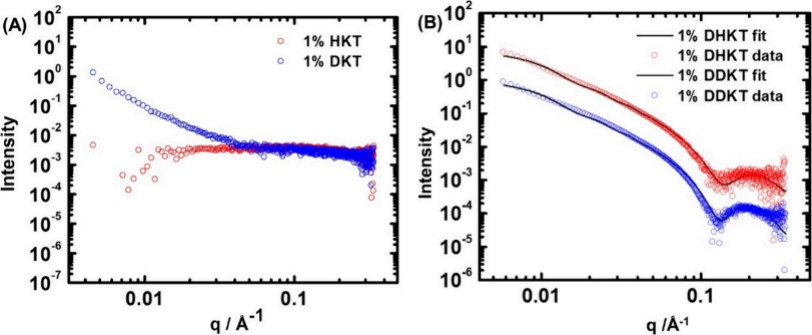
SAXS data for 1 wt % aqueous solution of (A) **HKT**, **DKT** and (B) **DHKT**, **DDKT**. Open symbols: measured data; lines fits as described in the text (fit parameters in SI Table S1). Only every 5th data point is shown for ease of visualization, and the data in (B) are offset vertically (×0.1 for **DDKT** data) for the same reason.

To complement cryo-TEM, SAXS experiments were performed. To obtain in situ information on the internal features of the nanostructures (shape and dimensions), synchrotron SAXS data were measured for the four cycloalkane-attached peptides under the same conditions as for the cryo-TEM images. From Figure 5, we can observe two data sets that are distinct from each other. The shapes of the intensity profiles for **HKT** and **DKT** are similar to each other and are different from those for **DHKT** and **DDKT,** which are similar to one another (Figure 5). **DKT** and especially **HKT** show very weak scattering that could not be fitted in a unique manner, although the upturn in the intensity at the low wavenumber *q* for **DKT** does indicate aggregation, although the structure is not well-defined (consistent with the above discussion of polydisperse globular structures revealed by cryo-TEM). In contrast, the SAXS data for **DHKT** and **DDKT** show well-defined form factor features consistent with extended nanotape-like structures based on bilayer arrangements of molecules. This is consistent with the cryo-TEM images in Figure 3C,D. As shown in Figure 5, the SAXS data can be well fitted using a form factor that represents the density profile across the bilayer as the sum of three Gaussians: one with a low electron density for the lipid core and two identical functions with a high electron density for the (cationic) peptide surfaces. This model has been used successfully by our group to describe the SAXS form factor of lipopeptide nanotapes.^64^ The fit parameters are listed in SI Table S1.

**Figure 6:**
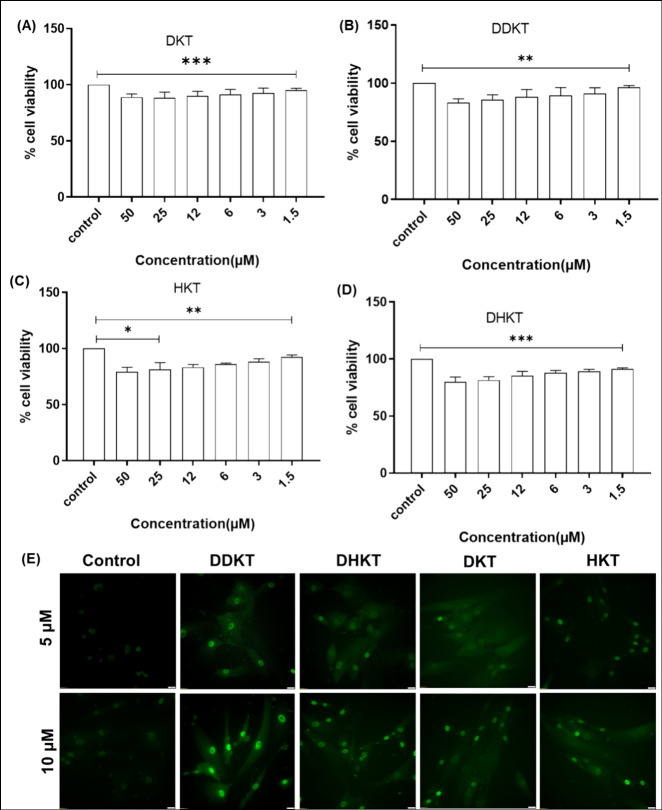
(A–D) Cell viability of cycloalkane peptides against HADF. The error bar corresponds to the standard deviation of the value from the mean (*n* = 3, *p < 0.05, **p < 0.01, ***p < 0.001, by performing two-tailed Student’s *t*- test). (E) Collagen deposition in HADF with dose-dependent peptide treatment. All experiments were conducted in triplicate. Scale bar = 100 μm.

Since KTTKS-based peptides show excellent collagen-stimulating properties, they have the potential as wound-healing biomaterials. This was examined for all four cycloalkane lipopeptides. To serve as practical systems for future applications in tissue engineering and regenerative medicine, lipopeptides must have good cytocompatibility. This was investigated via MTT assays to probe mitochondrial activity using HADF cells. For all the cycloalkane lipopeptides, the cell viability assay was performed up to 48 h of cell culture. The data in Figure 6 show that the cell viability is very high (there was no significant difference when compared with control cells) and the lipopeptides are nontoxic at concentrations as high as 50 μM (Figure 6A–D). Having established good cytocompatibility, a collagen deposition assay was performed, again using HADF cells. Treatment of cells with 5 or 10 μM of any of the lipopeptides increases the expression of collagen in comparison to controls (Figure 6E).

**Figure 7:**
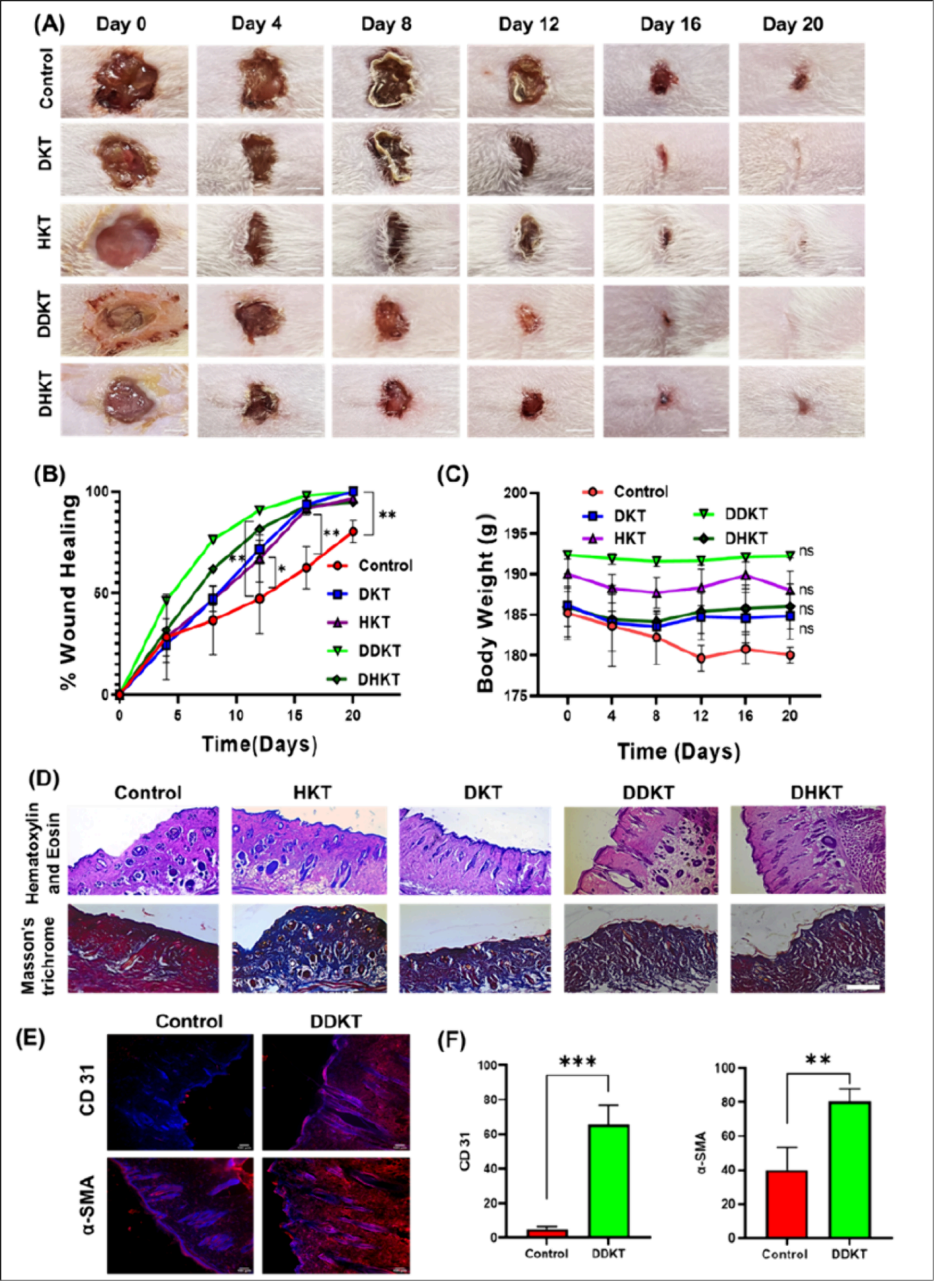
In vivo wound healing studied using a rat model: (A) Images of wounds treated with lipopeptides. The scale bar corresponds to 5 mm. (B) Development of wound healing. (C) Monitoring body weight. (D) Histological images of tissue slices stained with H&E or Masson’s trichrome staining to show collagen deposition in the wound healing process. (E) Representative images of immunofluorescence staining (CD31 and α-SMA) of 20-day rat wound sections. (F) Quantification of relative expression of the markers expressed in arbitrary units. Analysis and quantification of the fluorescence micrographs were done using ImageJ software. Experiments were performed in triplicate. Scale bar = 100 μm; ***p-value < 0.001.

These promising results motivated us to investigate the molecules’ potential to heal wounds in vivo using a male albino Wistar rat model. After injection of streptozotocin (45 mg/kg), rats consistently exhibited hyperglycemia within 3 weeks of diabetic induction. To determine whether treatment with solutions of the lipopeptides enhances wound closure in diabetic rats, wound closure rates (%) were calculated for each group. Following the creation of full-thickness skin lesions, no significant difference in wound closure was observed between the groups by day 4, other than in the **DDKT** treatment group. However, by days 8, 12, 16, and 20, wound closure was significantly accelerated in the **DDKT**- and **DKT**-treated groups compared to the PBS-treated controls (Figure 7A, B). Rats treated with the lipopeptide solutions exhibited rapid wound closure starting after day 4, achieving nearly 90% closure by day 20 post-injury. In contrast, PBS-treated diabetic control rats showed impaired wound closure (Figure 7A, B). Encouragingly, there were no statistically significant differences in body mass (Figure 7C) or blood glucose levels among the animals throughout the experimental period (*p* > 0.05).

Regeneration of hair follicles and blood vessels in the dermis are critical indicators of effective wound repair. Analysis of H&E-stained tissue images presented in Figure 7D shows that on day 20, while there was a significant difference between the control and treated groups in terms of epidermis and dermis regeneration, the **DDKT-** and **DKT-treated** groups also exhibited a significantly higher number of regenerated hair follicles, indicating a faster healing process for **DDKT** in particular, compared to the control group. During skin tissue repair, endothelial cells on the vascular wall grow into vascular buds along the basement membrane, maturing into microvessels and forming granulation tissue. On day 20, the number of blood vessels in **DDKT**-treated diabetic wounds was significantly higher than compared to control groups (Figure 7D). Masson’s trichrome staining was employed to evaluate collagen deposition in the wound periphery. As demonstrated in Figure 7D, the treatment group exhibited significantly higher collagen deposition (indicated by the blue stain). In contrast, the control group treated with PBS showed a predominance of thick wound scabs, which are stained red. By day 20, the **DDKT**-treated groups had developed a dense collagen network accompanied by a regenerated epidermis, while the control groups showed minimal recovery.

Immunofluorescence staining (Figure 7E) was conducted to evaluate neovascularization by targeting α-SMA (a marker for vascular smooth muscle cells) and CD31 (a marker specific to vascular endothelial cells). The data in Figure 7F illustrate that the expression levels of α-SMA and CD31 were significantly higher in the **DDKT** treatment group on day 20 compared to the untreated sections. These results suggest that **DDKT** exhibits a remarkable pro-vascularization capability. This effect is likely due to the angiogenic properties of the lipopeptide, supporting the three-dimensional formation of vascular networks, thereby enhancing nutrient delivery for epithelial regeneration, accelerating re-epithelialization, and significantly improving wound healing.

## Conclusions

In summary, the dicyclododecyl-KTTKS lipopeptide **DDKT** is a very promising lead candidate for wound healing applications as it stimulates significant wound healing, collagen production, and pro-angiogenic properties. All four lipopeptides show good cytocompatibility to dermal fibroblasts and dose-dependent collagen-stimulation properties, and **DDKT** and **DKT** show enhanced wound closure (4 days and later, following the application of solutions) in the diabetic rat model compared to controls. The enhanced wound healing for both **DDKT** and **DKT** suggests that it is the nature of the cycloalkane that influences bioactivity rather than the distinct self-assembly modes observed for double- compared to single-tailed molecules, possibly due to improved compatibility of the cyclododecyl lipid with cell membranes. It may be noted that the cyclododecyl chain length is closer to that of lipids in cell membranes, potentially improving the biocompatibility. However, the detailed mechanisms of uptake into cells and subsequent activity (neovascularization and collagen stimulation) require future investigation.

We are not aware of prior work on the self-assembly of cycloalkane lipopeptides, although crystal structures have been reported for short peptides with 1-aminocyclooctane-1-carboxylic acid or 1-aminocyclododecane-1-carboxylic acid side chains.^65,66^ Cycloalkyl-amino acids are incorporated into peptides due to the properties of conformationally constrained residues, although in our work the cycloalkyl units are used as hydrophobic tails in amphiphilic lipopeptides to drive self-assembly. The N-terminal cycloalkyl units can be compared in terms of hydrophobicity to certain bulky N-terminal units such as naphthalene widely used to modulate peptide self-assembly,^67^ although obviously not in terms of π–π interaction-driven aggregation.

Both double-tailed cycloalkane lipopeptides studied **DHKT** and **DDKT** self-assemble into nanosheet/nanotape structures based on extended β-sheet structures, whereas the single-chain cycloalkane lipopeptides form irregular globular structures with the peptide adopting a disordered conformation. The difference in the morphology between the double- and single-chained molecules reflects differences in the packing of the cycloalkane chains. Steric constraints for the double-chained molecules lead to packing in bilayer structures whereas the less bulky single-chained molecules can pack into globular structures.

## Supporting Information Available

HPLC and ESI–MS spectra for all cycloalkanes attached peptides, additional FTIR spectra, DLS data, and SAXS fit parameters (PDF)

## References

[ref1] Gazit E. Self-assembled Peptide Nanostructures: the Design of Molecular Building Blocks and Their Technological Utilization. Chem. Soc. Rev. 2007, 36 (8), 1263–1269. 10.1039/b605536m. 17619686

[ref2] Castelletto V.; Edwards-Gayle C. J.; Hamley I. W.; Barrett G.; Seitsonen J.; Ruokolainen J. Peptide-stabilized Emulsions and Gels from an Arginine-rich Surfactant-like Peptide with Antimicrobial Activity. ACS Appl. Mater. Interfaces 2019, 11 (10), 9893–9903. 10.1021/acsami.9b00581. 30785266 PMC7005944

[ref3] Vauthey S.; Santoso S.; Gong H.; Watson N.; Zhang S. Molecular Self-assembly of Surfactant-like Peptides to Form Nanotubes and Nanovesicles. Proc. Natl. Acad. Sci. U.S.A. 2002, 99 (8), 5355–5360. 10.1073/pnas.072089599. 11929973 PMC122773

[ref4] Adak A.; Mohapatra S.; Mondal P.; Jana B.; Ghosh S. Design of a Novel Microtubule Targeted Peptide Vesicle for Delivering Different Anticancer Drugs. Chem. Commun. 2016, 52 (48), 7549–7552. 10.1039/C6CC01769J. 27153208

[ref5] Haridas V. Tailoring of Peptide Vesicles: a Bottom-up Chemical Approach. Acc. Chem. Res. 2021, 54 (8), 1934–1949. 10.1021/acs.accounts.0c00690. 33823579

[ref6] Hamley I. W. Lipopeptides for Vaccine Development. Bioconjugate Chem. 2021, 32 (8), 1472–1490. 10.1021/acs.bioconjchem.1c00258. PMC8382226 34228433

[ref7] Shankar S. S.; Benke S. N.; Nagendra N.; Srivastava P. L.; Thulasiram H. V.; Gopi H. N. Self-assembly to Function: Design, Synthesis, and Broad spectrum Antimicrobial Properties of Short Hybrid E-vinylogous Lipopeptides. J. Med. Chem. 2013, 56 (21), 8468–8474. 10.1021/jm400884w. 24117107

[ref8] Vicente-Garcia C.; Colomer I. Lipopeptides as Tools in Catalysis, Supramolecular, Materials and Medicinal Chemistry. Nature Rev. Chem. 2023, 7 (10), 710–731. 10.1038/s41570-023-00532-8. 37726383

[ref9] Castelletto V.; Kaur A.; Kowalczyk R. M.; Hamley I. W.; Reza M.; Ruokolainen J. Supramolecular Hydrogel Formation in a Series of Self-assembling Lipopeptides with Varying Lipid Chain Length. Biomacromolecules 2017, 18 (7), 2013–2023. 10.1021/acs.biomac.7b00057. 28535062

[ref10] Hamley I. W. The Amyloid Beta Peptide: a Chemist’s Perspective. Role in Alzheimer’s and Fibrillization. Chem. Rev. 2012, 112 (10), 5147–5192. 10.1021/cr3000994. 22813427

[ref100] Hamley I. W. Peptide Nanotubes. Angew. Chem., Int. Ed. Engl. 2014, 53, 6866–6881. 10.1002/anie.201310006. 24920517

[ref11] Roberts K. D.; Azad M. A.; Wang J.; Horne A. S.; Thompson P. E.; Nation R. L.; Velkov T.; Li J. Antimicrobial Activity and Toxicity of the Major Lipopeptide Components of Polymyxin B and Colistin: Last-line Antibiotics against Multidrug-Resistant Gram-negative Bacteria. ACS Infect. Diseases 2015, 1 (11), 568–575. 10.1021/acsinfecdis.5b00085. 27525307 PMC4980087

[ref12] Dawgul M. A.; Greber K. E.; Bartoszewska S.; Baranska-Rybak W.; Sawicki W.; Kamysz W. In Vitro Evaluation of Cytotoxicity and Permeation Study on Lysine-and Arginine-based Lipopeptides with Proven Antimicrobial Activity. Molecules 2017, 22 (12), 2173 10.3390/molecules22122173. 29292739 PMC6150024

[ref13] Gouveia R. M.; Hamley I. W.; Connon C. J. Bio-fabrication and physiological self-release of tissue equivalents using smart peptide amphiphile templates. J. Mater. Sci.-Mater. Med. 2015, 26, 242 10.1007/s10856-015-5581-5. 26411438

[ref14] Rosa E.; De Mello L.; Castelletto V.; Dallas M. L.; Accardo A.; Seitsonen J.; Hamley I. W. Cell Adhesion Motif-functionalized Lipopeptides: Nanostructure and Selective Myoblast Cytocompatibility. Biomacromolecules 2023, 24 (1), 213–224. 10.1021/acs.biomac.2c01068. 36520063 PMC9832505

[ref15] Yang J.; Bahreman A.; Daudey G.; Bussmann J.; Olsthoorn R. C.; Kros A. Drug Delivery via Cell Membrane Fusion Using Lipopeptide Modified Liposomes. ACS Central Sci. 2016, 2 (9), 621–630. 10.1021/acscentsci.6b00172. PMC5043431 27725960

[ref16] Biswas A.; Chakraborty K.; Dutta C.; Mukherjee S.; Gayen P.; Jan S.; Mallick A. M.; Bhattacharyya D.; Sinha Roy R. Engineered Histidine-enriched Facial Lipopeptides for Enhanced Intracellular Delivery of Functional siRNA to Triple Negative Breast Cancer Cells. ACS Appl. Mater. Interfaces 2019, 11 (5), 4719–4736. 10.1021/acsami.8b13794. 30628773

[ref18] Hosseinkhani H.; Hong P.-D.; Yu D.-S. Self-assembled Proteins and Peptides for Regenerative Medicine. Chem. Rev. 2013, 113 (7), 4837–4861. 10.1021/cr300131h. 23547530

[ref19] Matson J. B.; Stupp S. I. Self-assembling Peptide Scaffolds for Regenerative Medicine. Chem. Commun. 2012, 48 (1), 26–33. 10.1039/C1CC15551B. PMC3355058 22080255

[ref20] Furth M. E.; Atala A.; Van Dyke M. E. Smart Biomaterials Design for Tissue Engineering and Regenerative Medicine. Biomaterials 2007, 28 (34), 5068–5073. 10.1016/j.biomaterials.2007.07.042. 17706763

[ref21] Kumar V. A.; Taylor N. L.; Shi S.; Wang B. K.; Jalan A. A.; Kang M. K.; Wickremasinghe N. C.; Hartgerink J. D. Highly Angiogenic Peptide Nanofibers. ACS Nano 2015, 9 (1), 860–868. 10.1021/nn506544b. 25584521 PMC4370274

[ref22] Taraballi F.; Sushnitha M.; Tsao C.; Bauza G.; Liverani C.; Shi A.; Tasciotti E. Biomimetic Tissue Engineering: Tuning the Immune and Inflammatory Response to Implantable Biomaterials. Adv. Healthcare Mater. 2018, 7 (17), 1800490 10.1002/adhm.201800490. 29995315

[ref23] Mardilovich A.; Craig J. A.; McCammon M. Q.; Garg A.; Kokkoli E. Design of a Novel Fibronectin-mimetic Peptide–amphiphile for Functionalized Biomaterials. Langmuir 2006, 22 (7), 3259–3264. 10.1021/la052756n. 16548586

[ref24] Graf J.; Ogle R. C.; Robey F. A.; Sasaki M.; Martin G. R.; Yamada Y.; Kleinman H. K. A Pentapeptide from the Laminin B1 Chain Mediates Cell Adhesion and Binds to 67000 Laminin Receptor. Biochemistry 1987, 26 (22), 6896–6900. 10.1021/bi00396a004. 2962631

[ref25] Aldag C.; Teixeira D. N.; Leventhal P. S. Skin Rejuvenation Using Cosmetic Products Containing Growth Factors, Cytokines, and Matrikines: A Review of the Literature. Clin., Cosmet. Invest. Dermatol. 2016, 9, 411–419. 10.2147/CCID.S116158. PMC5108505 27877059

[ref26] Jariwala N.; Ozols M.; Bell M.; Bradley E.; Bradley E.; Gilmore A.; Debelle L.; Sherratt M. J. Matrikines as Mediators of Tissue Remodelling. Adv. Drug Delivery Rev. 2022, 185, 114240 10.1016/j.addr.2022.114240. 35378216

[ref27] Mortazavi S. M.; Moghimi H. R. Skin Permeability, A Dismissed Necessity for Anti-Wrinkle Peptide Performance. Int. J. Cosmetic Sci. 2022, 44 (2), 232–248. 10.1111/ics.12770. 35302659

[ref28] Ferreira M. S.; Magalhaes M. C.; Sousa-Lobo J. M.; Almeida I. F. Trending Anti-Aging Peptides. Cosmetics 2020, 7 (4), 91 10.3390/cosmetics7040091.

[ref29] Ledwón P.; Errante F.; Papini A. M.; Rovero P.; Latajka R. Peptides as Active Ingredients: A Challenge for Cosmeceutical Industry. Chem. Biodivers. 2021, 18 (2), e2000833 10.1002/cbdv.202000833. 33348441

[ref30] Pelin J. N. B. D.; Lopes P. S.; Leite-Silva V.; Hamley I. W.; Andreo-Filho N., in preparation, 2024.

[ref31] Katayama K.; Armendarizborunda J.; Raghow R.; Kang A. H.; Seyer J. M. A Pentapeptide from Type-I Procollagen Promotes Extracellular-Matrix Production. J. Biol. Chem. 1993, 268 (14), 9941–9944. 10.1016/S0021-9258(18)82153-6. 8486721

[ref32] Lintner K. Compositions Containing Mixtures of Tetrapeptides and Tripeptides. 2003; patent WO/2005/048968.

[ref33] Jones R. R.; Castelletto V.; Connon C. J.; Hamley I. W. Collagen Stimulating Effect of Peptide Amphiphile C_16_–KTTKS on Human Fibroblasts. Mol. Pharmaceutics 2013, 10, 1063–1069. 10.1021/mp300549d. 23320752

[ref34] Castelletto V.; Hamley I. W.; Perez J.; Abezgauz L.; Danino D. Fibrillar Superstructure from Extended Nanotapes Formed by a Collagen-stimulating Peptide. Chem. Commun. 2010, 46, 9185–9187. 10.1039/c0cc03793a. 21031196

[ref35] Hamley I. W.; Dehsorkhi A.; Castelletto V. Coassembly in Binary Mixtures of Peptide Amphiphiles Containing Oppositely Charged Residues. Langmuir 2013, 29, 5050–5059. 10.1021/la400163q. 23534557

[ref36] Castelletto V.; Gouveia R. J.; Connon C. J.; Hamley I. W. New RGD- Peptide Amphiphile mixtures Containing a Negatively Charged Diluent. Faraday Discuss. 2013, 166, 381–397. 10.1039/c3fd00064h. 24611289

[ref37] Matson J. B.; Zha R. H.; Stupp S. I. Peptide Self-assembly for Crafting Functional Biological Materials. Cur. Opin. Solid State Mater. Sci. 2011, 15, 225–235. 10.1016/j.cossms.2011.08.001. PMC3224089 22125413

[ref38] Boekhoven J.; Stupp S. I. 25th Anniversary Article: Supramolecular Materials for Regenerative Medicine. Adv. Mater. 2014, 26, 1642–1659. 10.1002/adma.201304606. 24496667 PMC4015801

[ref39] Arslan E.; Garip I. C.; Gulseren G.; Tekinay A. B.; Guler M. O. Bioactive Supramolecular Peptide Nanofibers for Regenerative Medicine. Adv. Healthcare Mater. 2014, 3 (9), 1357–1376. 10.1002/adhm.201300491. 24574311

[ref40] Guler M. O.; Hsu L.; Soukasene S.; Harrington D. A.; Hulvat J. F.; Stupp S. I. Presentation of RGDS Epitopes on Self-assembled Nanofibers of Branched Peptide Amphiphiles. Biomacromolecules 2006, 7 (6), 1855–1863. 10.1021/bm060161g. 16768407 PMC2547993

[ref41] Peypoux F.; Bonmatin J. M.; Wallach J. Recent Trends in the Biochemistry of Surfactin. Appl. Microbiol. Biotechnol. 1999, 51, 553–563. 10.1007/s002530051432. 10390813

[ref42] Bonmatin J. M.; Laprevote O.; Peypoux F. Diversity Among Microbial Cyclic Lipopeptides: Iturins and Surfactins. Activity-structure Relationships to Design New Bioactive Agents. Comb. Chem. High Throughput Screen 2003, 6 (6), 541–556. 10.2174/138620703106298716. 14529379

[ref43] Ongena M.; Jacques P. Bacillus Lipopeptides: Versatile Weapons for Plant Disease Biocontrol. Trends Microbiol. 2008, 16 (3), 115–125. 10.1016/j.tim.2007.12.009. 18289856

[ref44] Hamley I. W.; Dehsorkhi A.; Jauregi P.; Seitsonen J.; Ruokolainen J.; Coutte F.; Chataigné G.; Jacques P. Self-assembly of Three Bacterially-derived Bioactive Lipopeptides. Soft Matter 2013, 9, 9572–9578. 10.1039/c3sm51514a. 26029764

[ref45] Hamley I. W. Lipopeptides: from Self-assembly to Bioactivity. Chem. Commun. 2015, 51, 8574–8583. 10.1039/C5CC01535A. 25797909

[ref46] Murakami Y.; Nakano A.; Yoshimatsu A.; Uchitomi K.; Matsuda Y. Characterization of Molecular Aggregates of Peptide Amphiphiles and Kinetics of Dynamic Processes Performed by Single-Walled Vesicles. J. Am. Chem. Soc. 1984, 106 (12), 3613–3623. 10.1021/ja00324a034.

[ref47] Mardilovich A.; Kokkoli E. Biomimetic Peptide-amphiphiles for Functional Biomaterials: The Role of GRGDSP and PHSRN. Biomacromolecules 2004, 5, 950–957. 10.1021/bm0344351. 15132686

[ref48] Cavalli S.; Handgraaf J. W.; Tellers E. E.; Popescu D. C.; Overhand M.; Kjaer K.; Vaiser V.; Sommerdijk N.; Rapaport H.; Kros A. Two-dimensional Ordered Beta-Sheet Lipopeptide Monolayers. J. Am. Chem. Soc. 2006, 128 (42), 13959–13966. 10.1021/ja065479v. 17044724

[ref49] Craig J. A.; Rexeisen E. L.; Mardilovich A.; Shroff K.; Kokkoli E. Effect of Linker and Spacer on the Design of a Fibronectin-mimetic Peptide Evaluated via Cell Studies and AFM Adhesion Forces. Langmuir 2008, 24 (18), 10282–10292. 10.1021/la702434p. 18693703

[ref50] Dasgupta A. Exploring Architectures at the Nanoscale: the Interplay between Hydrophobic Twin Lipid Chains and Head Groups of Designer Peptide Amphiphiles in the Self-assembly Process and Application. Soft Matter 2016, 12 (19), 4352–4360. 10.1039/C6SM00147E. 27079384

[ref51] Dasgupta A.; Das D. Designer Peptide Amphiphiles: Self-Assembly to Applications. Langmuir 2019, 35 (33), 10704–10724. 10.1021/acs.langmuir.9b01837. 31330107

[ref52] Hamley I. W.; Kirkham S.; Dehsorkhi A.; Castelletto V.; Reza M.; Ruokolainen J. Toll-like Receptor Agonist Lipopeptides Self-Assemble into Distinct Nanostructures. Chem. Commun. 2014, 50, 15948–15951. 10.1039/C4CC07511K. 25382300

[ref53] Lu B. L.; Williams G. M.; Brimble M. A. TLR2 Agonists and Their Structure–Activity Relationships. Org. Biomol. Chem. 2020, 18, 5073–5094. 10.1039/D0OB00942C. 32582902

[ref54] Manoj K. M.; Jayakumar R.; Rakshit S. K. Physicochemical Studies on Reverse Micelles of Sodium bis(2-ethylhexyl) sulfosuccinate at Low Water Content. Langmuir 1996, 12 (17), 4068–4072. 10.1021/la950279a.

[ref55] Gasymov O. K.; Glasgow B. J. ANS fluorescence: Potential to Augment the Identification of the External Binding Sites of Proteins. Biochim. Biophys. Acta-Proteins and Proteomics 2007, 1774 (3), 403–411. 10.1016/j.bbapap.2007.01.002. PMC2039916 17321809

[ref56] Adak A.; Castelletto V.; de Sousa A.; Karatzas K.-A.; Wilkinson C.; Khunti N.; Seitsonen J.; Hamley I. W. Self-assembly and Antimicrobial Activity of Lipopeptides Containing Lysine-rich Tripeptides. Biomacromolecules 2024, 25 (2), 1205–1213. 10.1021/acs.biomac.3c01184. 38204421 PMC10865344

[ref57] Hamley I. W. Peptide Fibrillization. Angew. Chem., Int. Ed. Engl. 2007, 46 (43), 8128–8147. 10.1002/anie.200700861. 17935097

[ref58] Levine H. Thioflavine T Interaction with Synthetic Alzheimer’s Disease β-amyloid Peptides: Detection of Amyloid Aggregation in Solution. Protein Sci. 1993, 2 (3), 404–410. 10.1002/pro.5560020312. 8453378 PMC2142377

[ref59] Woody R. W. Circular Dichroism of Peptides and Proteins. In Circular Dichroism. Principles and Applications, Nakanishi K.; Berova N.; Woody R. W., Eds. VCH: New York, 1994; pp 473–496.

[ref60] Kelly S. M.; Jess T. J.; Price N. C. How to Study Proteins by Circular Dichroism. Biochim. Biophys. Acta-Proteins and Proteomics 2005, 1751, 119–139. 10.1016/j.bbapap.2005.06.005. 16027053

[ref61] Bulheller B. M.; Rodger A.; Hirst J. D. Circular and Linear Dichroism of Proteins. Phys. Chem. Chem. Phys. 2007, 9 (17), 2020–2035. 10.1039/b615870f. 17464384

[ref62] Barth A. Infrared Spectroscopy of Proteins. Biochim. Biophys. Acta-Bioenergetics 2007, 1767 (9), 1073–1101. 10.1016/j.bbabio.2007.06.004. 17692815

[ref63] Jackson M.; Mantsch H. H. The Use and Misuse of FTIR Spectroscopy in the Determination of Protein Structure. Crit. Rev. Biochem. Mol. Biol. 1995, 30 (2), 95–120. 10.3109/10409239509085140. 7656562

[ref64] Castelletto V.; Hamley I. W. Methods to Characterize the Nanostructure and Molecular Organization of Amphiphilic Peptide Assemblies. Methods Mol. Biol. 2018, 1777, 3–21. 10.1007/978-1-4939-7811-3_1. 29744826

[ref65] Datta S.; Rathore R. N. S.; Vijayalakshmi S.; Vasudev P. G.; Rao R. B.; Balaram P.; Shamala N. Peptide Helices with Pendant Cycloalkane Rings.: Characterization of Conformations of 1-aminocyclooctane-1-carboxylic acid (Ac_8_c) Residues in Peptides. J. Pept. Sci. 2004, 10 (3), 160–172. 10.1002/psc.507. 15113088

[ref66] Vasudev P. G.; Aravinda S.; Shamala N. Crystal Structure of a Tripeptide Containing Aminocyclododecane Carboxylic Acid: a Supramolecular Twisted Parallel -sheet in Crystals. J. Pept. Sci. 2016, 22 (3), 166–173. 10.1002/psc.2854. 26856690

[ref67] Hamley I. W. Self-Assembly, Bioactivity and Nanomaterials Applications of Peptide Conjugates with Bulky Aromatic Terminal Groups. ACS Appl. Bio. Mater. 2023, 6, 384–409. 10.1021/acsabm.2c01041. PMC9945136 36735801

