# Supplementary material for: Self-Assembly and Wound Healing Activity of Biomimetic Cycloalkane-Based Lipopeptides
Source: ACS Appl Mater Interfaces. 2024 Oct 18;16(43):58417–26. doi: 10.1021/acsami.4c14162 (PMC11533170; doi:10.1021/acsami.4c14162)
Supplement: Supplementary file 1 — am4c14162_si_001.pdf [file am4c14162_si_001.pdf]

Supporting Information

Self-assembly and Wound Healing Activity of Biomimetic Cycloalkane-Based Lipopeptides

Anindaysundar Adak,[a] Valeria Castelletto, [a] Ian W. Hamley,[a,*] Jani Seitsonen,[b] Aniket Jana,[c] Satyajit Ghosh,[c] Nabanita Mukherjee, [c] Surajit Ghosh[c,d]

[a] *School of Chemistry, Pharmacy and Food Biosciences, University of Reading, Whiteknights, Reading RG6 6AH, U.K.*

[b] *Nanomicroscopy Center, Aalto University, Puumiehenkuja 2, FIN-02150 Espoo, Finland*

[c] *Smart Healthcare, Interdisciplinary Research Platform, Indian Institute of Technology, Jodhpur, Rajasthan 342030, India*

[d] *Department of Bioscience and Bioengineering, Indian Institute of Technology, Jodhpur, Rajasthan, 342030, India*

[*] Author for correspondence. I.W.Hamley@reading.ac.uk

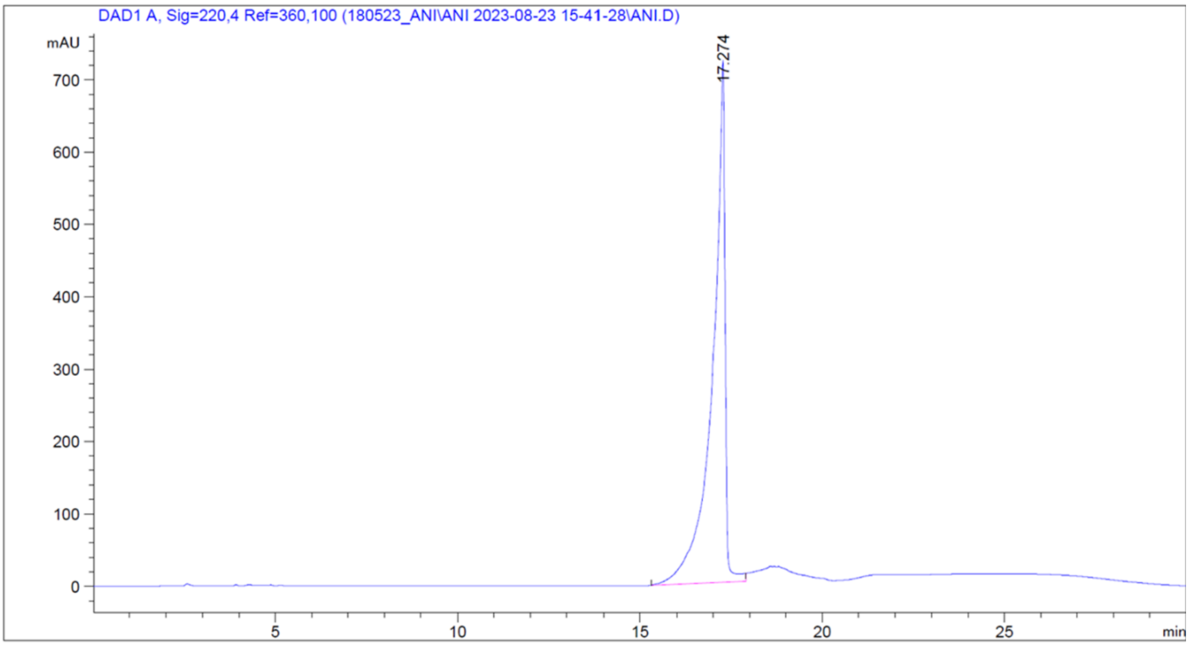

Peak #	RetTime [min]	Sig	Type	Area [mAU*s]	Height [mAU]	Area %
1	17.274	1	BB	1.71157e4	722.15924	100.0000
Totals :				1.71157e4	722.15924	

Figure S1. HPLC chromatogram of single cyclododecane- KTTKS (**DKT**).

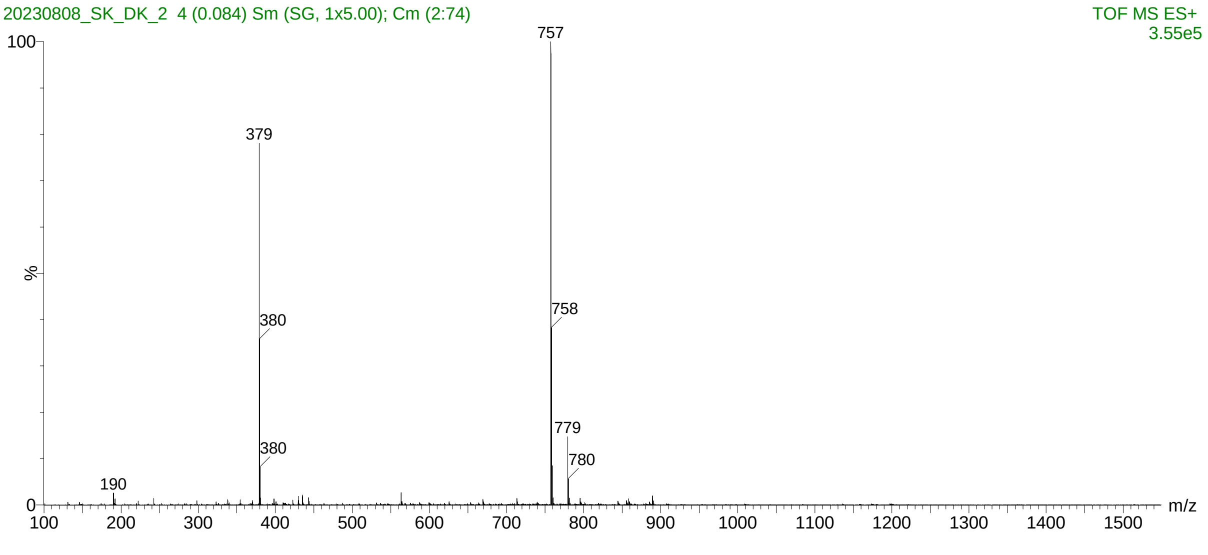

Figure S2. ESI-MS data of **DKT** (M = 757 g mol^{-1}).

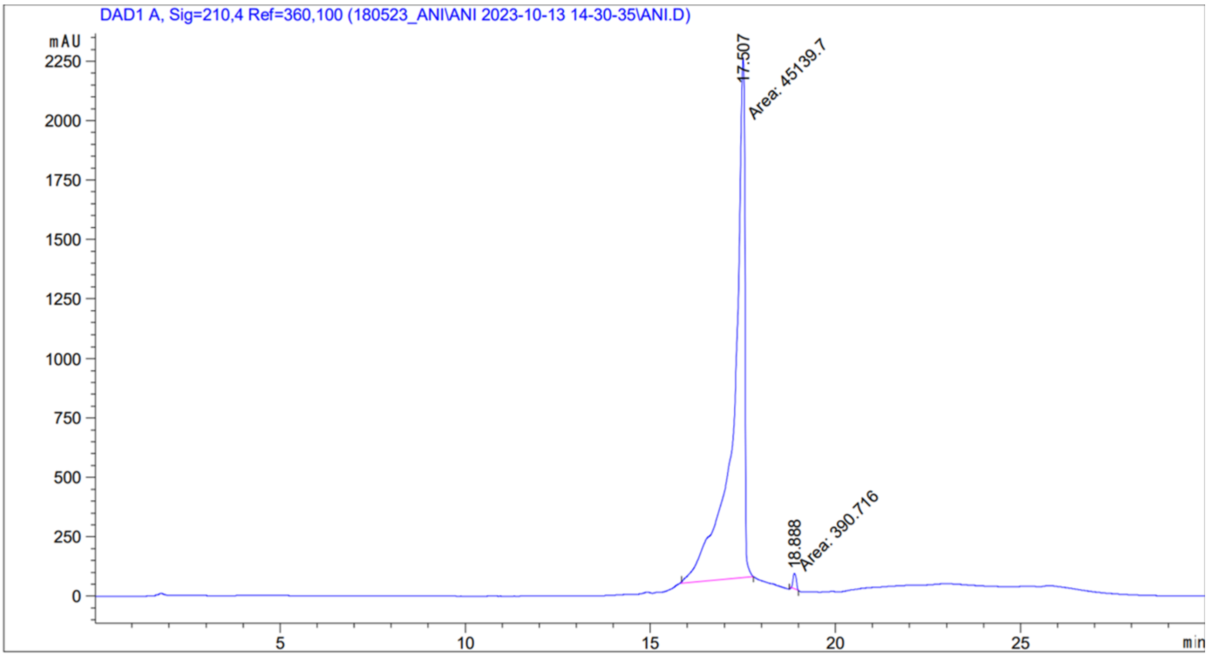

Peak #	RetTime [min]	Sig	Type	Area [mAU*s]	Height [mAU]	Area %
1	17.507	1	MM T	4.51397e4	2177.81348	99.1419
2	18.888	1	MM T	390.71582	64.79750	0.8581

Figure S3. HPLC chromatogram of double cyclododecane-KTTKS (**DDKT**).

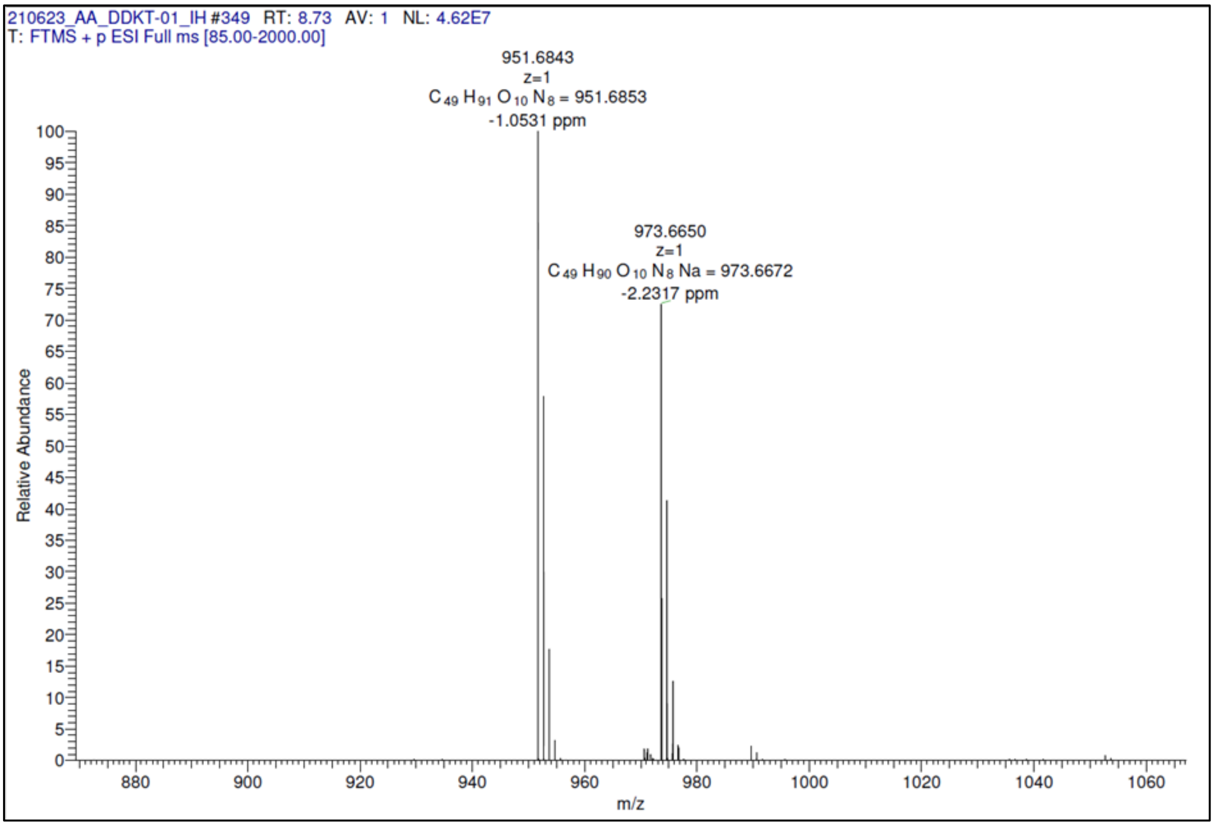

Figure S4. ESI-MS data of **DDKT** (M = 951.6 g mol^{-1}, M+Na^{+} = 973.6 g mol^{-1}).

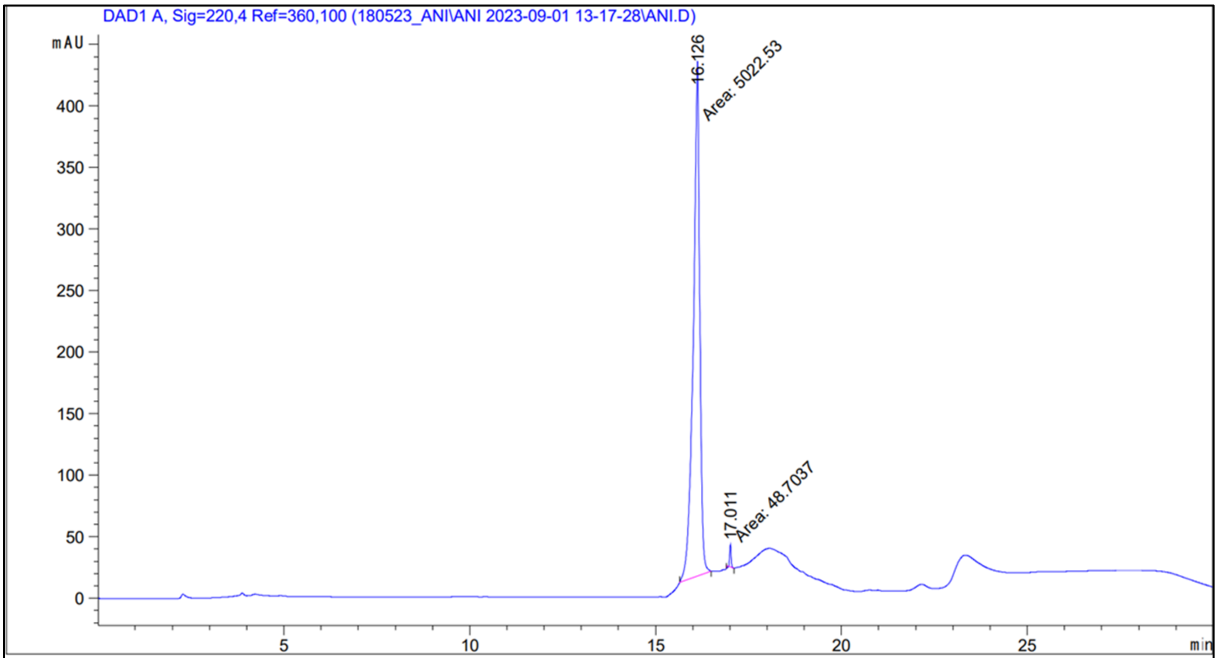

Peak #	RetTime [min]	Sig	Type	Area [mAU*s]	Height [mAU]	Area %
1	16.126	1	MM T	5022.52637	419.56595	99.0396
2	17.011	1	MM T	48.70371	17.95663	0.9604
Totals :				5071.23008	437.52258	

Figure S5. HPLC chromatogram of single cycloheptyl- KTTKS (**HKT**).

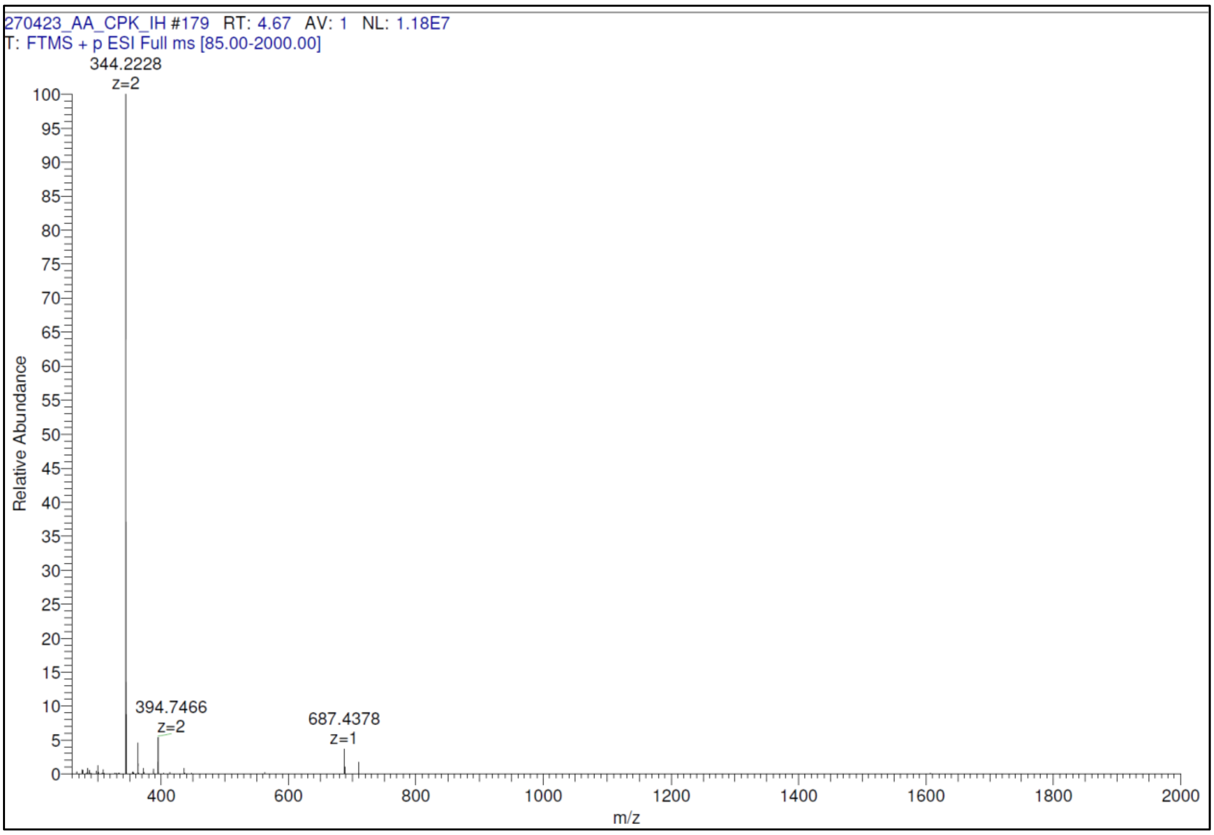

Figure S6. ESI-MS data of **HKT** (M = 687.4 g mol^{-1}, M^{+}/2 = 344.2 g mol^{-1}).

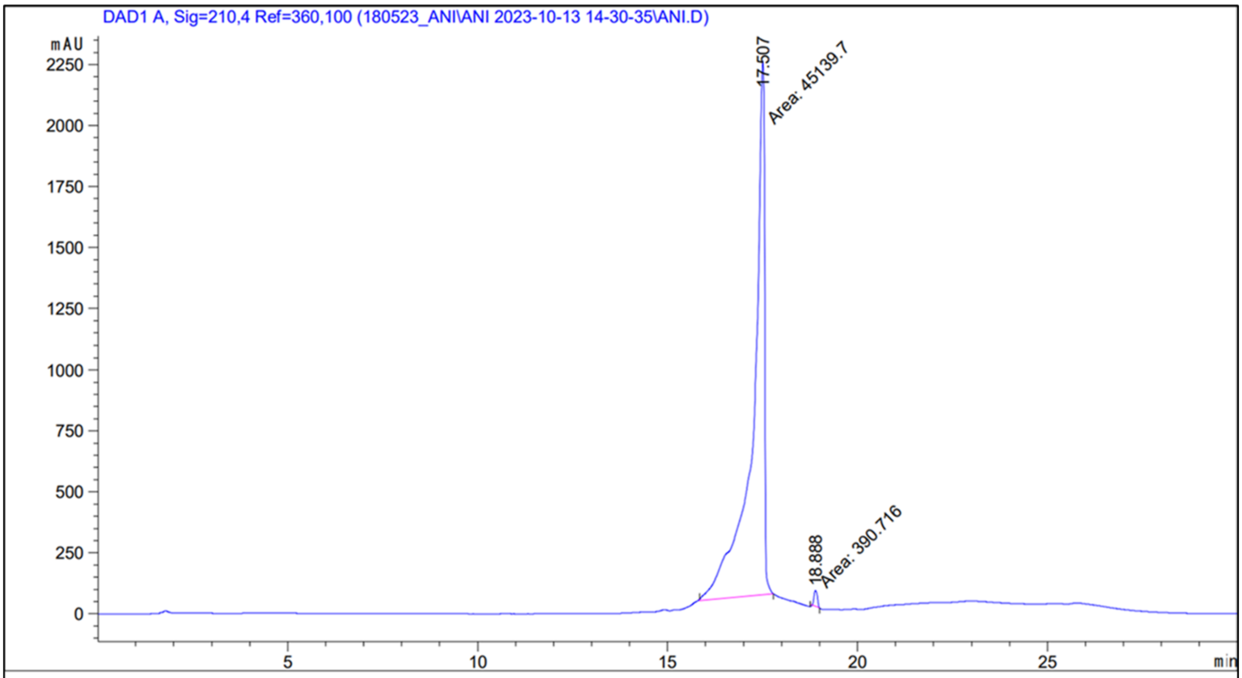

Peak #	RetTime [min]	Sig	Type	Area [mAU*s]	Height [mAU]	Area %
1	17.507	1	MM T	4.51397e4	2177.81348	99.1419
2	18.888	1	MM T	390.71582	64.79750	0.8581

Figure S7. HPLC chromatogram of double cycloheptyl- KTTKS (**DHKT**).

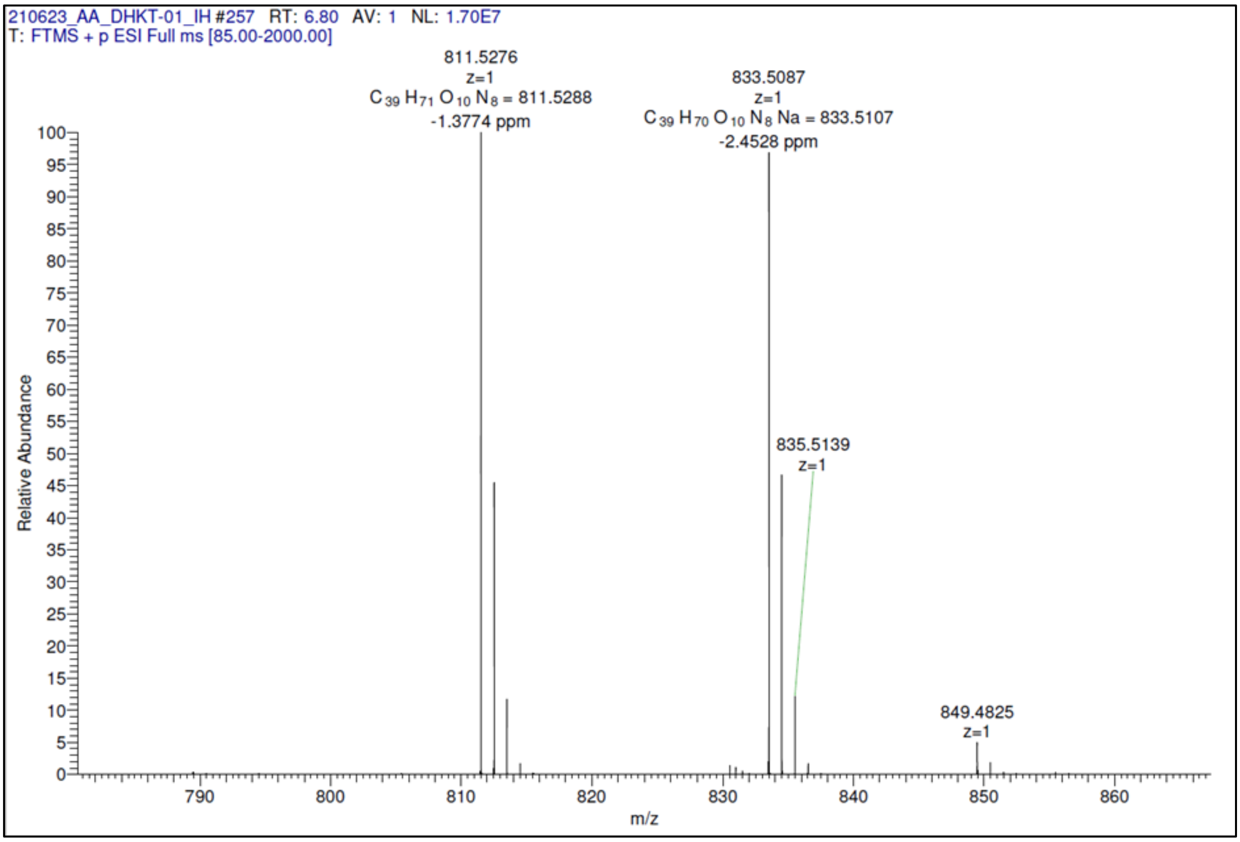

Figure S8. ESI-MS data of **DHKT** (M = 811.5 g mol^{-1}, M+Na^+ = 833.5 g mol^{-1}).

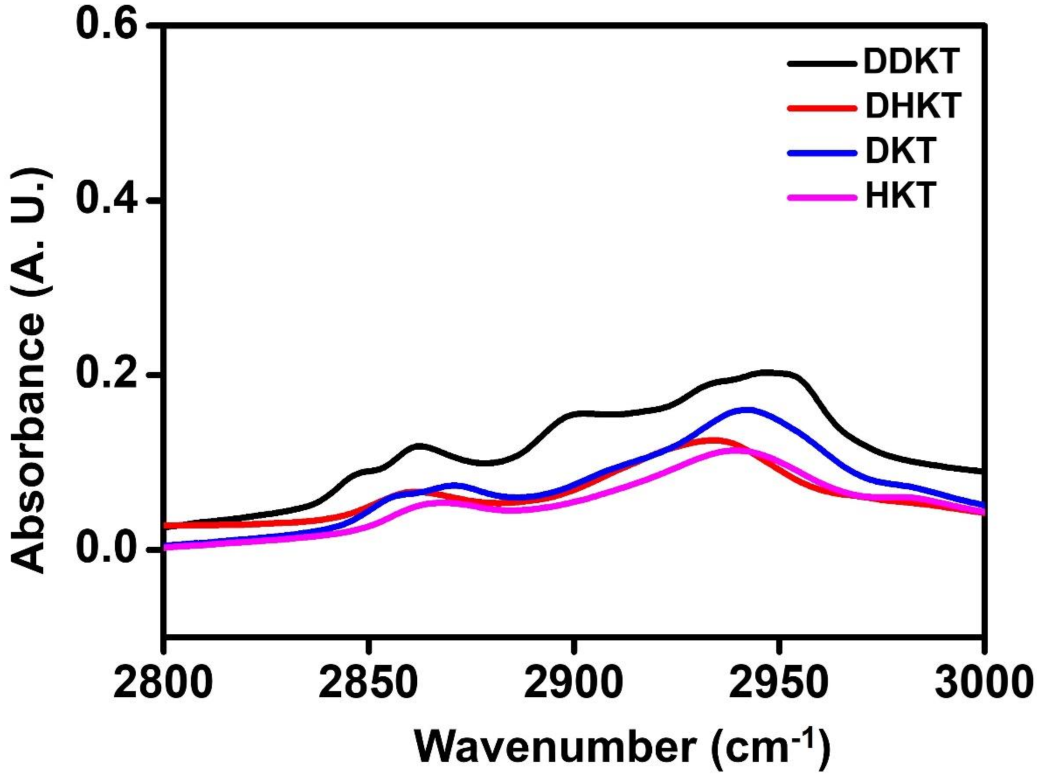

Figure S9. Cycloalkane ring vibrational band region of FTIR spectra of the four lipopeptides.

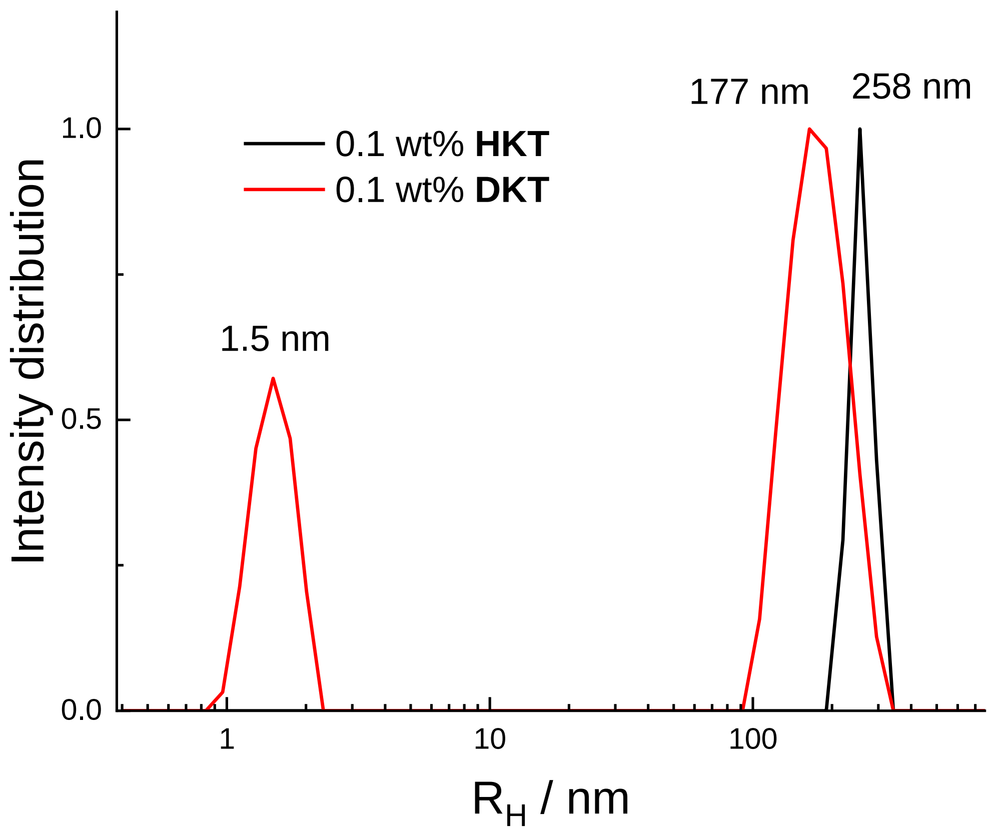

Figure S10. Distributions of hydrodynamic radius from DLS data.

Table S1. Parameters extracted from the fitting of the SAXS data for 1 wt% solutions at pH 8 using a Gaussian bilayer model.[a]

	DHKT	DDKT
$t \pm \Delta t$ [Å]	21.0± 3.0	23.5± 3.0
η_{out} [cm^{-1}]	6.22×10^{-7}	6.50×10^{-7}
σ_{out} [Å][b]	5.0	5.0
η_{in} [cm^{-1}]	-1.25×10^{-7}	-1.74×10^{-8}
σ_{in} [Å][b]	5.0	5.0
D [Å][b]	500	500
C [cm^{-1}]	3.32×10^{-4}	8×10^{-4}

[a] Data fitted using the software SASfit.[1-2]

[b] Fixed Parameter

Key: layer thickness t (Gaussian polydispersity Δt), scattering contrast of outer layers η_{out}, and inner layer η_{in}, Gaussian widths σ_{in} and σ_{out} of inner and outer layers respectively, diameter (width) of layer system D (when $D >> t$ as here, it acts as a scaling parameter for the form factor). **Background:** constant background, C.

References

(1) Bressler, I.; Kohlbrecher, J.; Thünemann, A. F., SASfit: a tool for small-angle scattering data analysis using a library of analytical expressions. *J. Appl. Cryst.* **2015,** *48*, 1587-1598.

(2) Kohlbrecher, J.; Bressler, I., Updates in SASfit for fitting analytical expressions and numerical models to small-angle scattering patterns. *J.Appl. Cryst.* **2022,** *55*, 1677-1688.
